# American Medical Association

**Published:** 1855-06

**Authors:** 


### MEDICINE AND SURGERY.

JUNE, 1855. NEW SERIES. VOL. III.—NO. 6.

### ART. I.—AMERICAN MEDICAL ASSOCIATION. PROCEEDINGS OF THE EIGHTH ANNUAL MEETING, HELD IN PHILADELPHIA, PA., MAY 1, 2, 3, 4, 1855.

REPORTED FOR THE NEW JERSEY MEDICAL REPORTER.

ON the morning of Tuesday, May 1st, at 11 o'clock, the Association assembled in the spacious hall of the Musical Fund building, and at 11½ A. M., was called to order by the President, Dr. C. A. POPE, of St. Louis, the Vice Presidents taking their seats on either hand of the President. The ex-Presidents were invited to take seats on the platform.

Drs. E. S. LEMOINE, of Missouri, and FRANCIS WEST, of Penn., took their seats as Secretaries.

Dr. ISAAC HAYS, of Philad., Chairman of the Committee of Arrangements, on behalf of the Profession of Philadelphia, extended a cordial greeting to the members of the Association. Dr. HAYS then stated that three hundred and thirty-seven delegates had registered their names.

The Chair announced the business first in order, to be the calling of the roll.

It was suggested that each delegate should rise to his feet as his name was called, which was approved.

The following are the States represented and the number of delegates from each:—

Maine 8; New Hampshire 7; Vermont 4; Massachusetts 53; Rhode Island 7; Connecticut 31; New York 76; New Jersey 29; Pennsylvania 124; Delaware 11; Maryland 30; District of Columbia 12; Virginia 18; North Carolina 5; South Carolina 17; Georgia 1; Alabama 4; Tennessee 4; Kentucky 8; Ohio 26; Indiana 5; Illinois 8; Michigan 6; Iowa 1; Wisconsin 5; Missouri 5; U. S. Army 1; U. S. Navy 3; Parisian Med. Society 1. Total number of delegates, 510.

The President, Dr. C. A. POPE, being called upon, delivered the annual address, as follows:—

*Gentlemen:* With feelings of grateful pleasure, I meet you, and greet you, on this occasion.

For high and useful purposes, have we assembled from the wide extent of our beloved country. The elevation of a noble profession—the promotion of science—the good of humanity—these have been, are, and will continue to be, the objects of our Association. Whether we have, thus far, done much or little, our sole aim has been the advancement of the best interests of our fellow men. I shall not assert that we have done as much as we might have done, or that the course hitherto pursued by us, is so perfect, as to admit of no improvement. Were such the fact, and were the Association a firmly established institution, I might have experienced more hesitation in the selection of a theme for the present occasion. And since we cannot, as yet, I think, urge such a claim, the few suggestions which I shall offer, are made with becoming diffidence, but at the same time with a deep sense of their importance to the welfare and perpetuity of our Association.

Some strictures on our proceedings, in medical and other journals, have appeared within the last year, as well as in previous years. I shall not here blame the authors of them. They are, doubtless, as proud of our noble profession as we, and equally with us, anxious for the advancement of its interests and its honor. I thank them for their suggestions. All of us are ready to hear them and to profit by them. If any more effectual mode of arriving at truth can be devised, than that which we have heretofore pursued, all of us are ready to follow it, and would rather thank than quarrel with those who may propose it.

Physicians have an almost superhuman mission to fulfil. The goal of their ambition, and their hopes, and their duty, stands at the *ultima thule* of human capacity—nay, rather beyond it. It cannot, indeed, be said, that their duties are beyond their powers, but their ambition, their hopes, their wishes certainly are. They would gladly know, not only all the secrets of organization, but those also of physiology, pathology, and therapeutics. To arrive at such knowledge, is, perhaps, beyond the attainment of the human mind. Multiform are the elements which enter into the problem of health and disease. Health, is, itself, a constant change of composition—diseases are ever-varying changes, supervening on this.

Do we not know, with all our advancement, and after all the toil of our predecessors for two thousand years, the exact changes in which any disease, the fevers for instance, consists? And even when we shall have learned these, so as to understand them as well as the most ordinary chemical changes, the ever-varying character of most diseases, and the inward disturbing influences upon them, of the mental and moral emotions, would require to follow them a continued stretch and power of intellect of which it is doubtful if man be capable. This exactness of knowledge is not, I grant, necessary to the very successful practice of medicine. Our profession can render great and important services to man without it, but with it, it would be still more serviceable. To it our ambition tends. To this perfect knowledge we aspire. Although we may never reach, we can yet eternally approach it. In the vast region of our researches, there is no probability that human genius will ever, Alexander like, weep for the want of unconquered provinces. Beyond the conquests of the future heroes of the profession, there will always be a boundless field for the ambitious and philanthropic explorer. In the language of a western student, "the science of medicine, like the liver of Prometheus, is sufficient to glut the eagles of all time."

The object of this Association is to do something to advance the profession towards the far-distant goal of perfection—to aid the solution of some of the problems and enigmas of life and organization—to add some material to the growing temple, whose foundations were so firmly laid by the Coan sage—and to do its part, as best it may, in the cause of humanity. Nor do I think, that so far, it has altogether failed. Many valuable contributions to science have been elicited—professional ambition has been stimulated—an *esprit-de-corps* has been successfully evoked and established. The strength of the profession has acquired additional power by the union of its members. This Association has been to physicians, what the railroad and electric wires are to commerce, and the interchange of useful knowledge to States and nations. It has made us one, and as I have just remarked, in unity there is power. This association has stimulated thought. Chaotic and void would forever remain the masses of facts accumulated by the observations of ages, but for the co-ordinating and logical power of reason. It sits in judgment on the silent phenomena, as a "refiner of gold, and a purifier of silver." It forces the voiceless facts to mount the tripod of the oracle, and speak forth words of wisdom. The scalpel, the crucible, the microscope, may be subsidiary to its purposes and ends, but they cannot supply its place. Fixed and patient thought, in medicine, as in the other departments of science, is the Aladdin's lamp that lights the footsteps of the discoverer. To stimulate attention and thought is to accelerate many a new discovery—to hasten the advent and establishment of important principles yet in the womb of the future. May not our Association do this more effectually than it has hitherto done?

Let all the contributions be read and attentively considered. Such a course would certainly be more encouraging, as well as more respectful, to their authors. Let the reports be deliberately and fully discussed, and let them go forth to the world with the sanction or criticisms of the Association. This would require time, it is true, but if we have time to meet at all, surely a few days would make but little difference. The good that would be effected would yield a tenfold compensation for the time employed. Every one must admit that three or four days is too short a time for the Association rightly to fulfil its annual mission.

I would, moreover, respectfully suggest that time be taken for the discussion of some of the leading topics of medical philosophy. Amongst these, may be mentioned the nature, causes, and treatment of cholera, yellow fever, &c.,—hygiene, and the laws of health affecting masses of men—quarantine—the causes of mortality among children—the chemical and vital doctrines of life. Questions like these, indicated a year in advance for discussion, would excite a carefulness of investigation, and a degree of attention and thought which could not fail to clear away much of the darkness and doubt in which they are yet shrouded. Nothing so sharpens the intellectual powers as public debate. It fixes attention, and strains to the utmost every faculty. I have no hesitation in saying that facts enough have been accumulated to establish great and general principles, of which the medical world is yet in ignorance or doubt. Nothing would contribute more to demonstrate these principles than the collision of matured intellects in public debate. What a mass of facts, and argument, and demonstration would be brought to bear, on any of the subjects alluded to, if some of the best minds in the profession were to debate them after a year's preparation! Observed facts are the crude materials of science—the intellect is the master builder of its august temple. I make these suggestions for your consideration. All the scientific meetings in this country and in Europe, employ more time than ours has hitherto employed. Evidently we must protract our sessions, if we would render them as serviceable to science as they may be. No member of the Association will be required to remain longer than suits his wishes or convenience. Some fifty or sixty, more or less, would always be found to listen with eagerness to scientific papers, and engage with pleasure in scientific discussions.

The time has probable arrived, for a change in our plan of organization, which will admit of the selection of a permanent place for the future meetings of the Association. There are evident advantages incident to both the migratory and stationary plans. These might, perhaps, be easily reconciled and secured. A proposition, if I mistake not, was made some years ago, by the Smithsonian Institution, and I would respectfully suggest, whether it would not be in accordance with the best interests of the Association, to hold biennial meetings in Washington, and the alternate ones, as now, at different points of our common country. We might thus secure all the advantages of a fixed abode, in the way of preserving the archives, making collections, etc., whilst by meeting in various localities, we could not fail to excite that wide-spread interest among the profession, and obtain such accessions of new members as would greatly enhance the high and useful objects of our Association. Should this proposal meet with your approbation, I would further intimate that policy would perhaps require the meetings of the Association at the National Capitol, to be held in the years of the short sessions of Congress.

I shall say but little of the legislative duties of the Association. I shall say nothing of the propriety or impropriety of getting laws passed to regulate the practice of medicine, and furnish standards for candidates for the Doctorate. Perhaps the Association can do but little in this respect. Ours is a popular government, and the people are disposed to allow the largest freedom in everything pertaining to medicine, medical schools, and physicians. Laws passed against quackery one year, are revoked the next. Our country is the paradise of quacks. All good things have their attendant evils, and this unbridled liberty is one of the evils of a popular govermnent. May we not hope, however, that even this evil may disappear, as general education and the cultivation of the masses advance? At any rate the people are not yet disposed to put down the quacks, nor to require too high a degree of qualification for those of the regular profession. After all, laws can make only mediocre physicians. They can require the candidates to know only so much—to be qualified to a certain degree; and this degree will always be far lower than that to which the true lovers of knowledge would attain, without any legislation on the subject. The greater lights of the profession cannot be manufactured after any process of legislative enactment. Thirst of knowledge, self-love, philanthropy, burning ambition—these make the great physician and surgeon. These have made all the worthies of the past—not legislation. Legislation cannot drive the drone to the proud heights of professional eminence. When these heights are reached, it will be seen that the successful aspirant has been stimulated by a stronger power.

To him the laurel blossoms of renown and the life-giving mission of his art, are dearer and more attractive than was the mystic bough of the sibyl to the eager Æneas, or, than the golden apples, guarded by sleepless dragons, to the Hesperian daughters.

Whatever course you may think proper to pursue, I am sure that your objects will be, the advancement of science—the good of mankind—the honor and glory of the profession. We have the dignity and character of a noble calling to sustain—of a profession which has numbered, for two thousand years and more, some of the wisest and best men in all countries and all times. It is no trivial matter to sustain the rank and respectability of a vocation which can boast of a Hippocrates, a Harvey, a Hunter, of the most erudite and beneficent of sages and philanthropists the world ever saw—of a profession which has furnished to every nation its *clarum et venerabile nomen.*

On the eve of the battle of the pyramids, Napoleon exclaimed, Soldiers! from the height of yon monuments forty centuries look down upon you. Gentlemen, from the heights of past ages, countless worthies of our God-like profession point and beckon to a goal more elevated than that which attracts legislators and conquerors, Solons and Cæsars.

On motion of Dr. J. B. BIDDLE,

*Resolved*, That the thanks of the Association are unanimously tendered to the President, for the able and eloquent address just delivered; also, that a copy be obtained for publication.

Dr. HAYS stated that, in accordance with the plans of the Committee of Arrangements, it would be necessary for the Convention to hold its sessions from 9 A. M. to 3 P. M., with one hour recess each day, which, on motion, was agreed to.

A recess of fifteen minutes was now declared, to allow the assembling of the various State delegations for the purpose of appointing one member from each to serve in a committee to report nominations for permanent officers of the Association.

On the re-assembling of the Convention, Dr. N. S. DAVIS, Vice President, in the chair, Dr. D. FRANCIS CONDIE moved that all permanent members who, through want of notification, had not paid their assessment, but who had subsequently paid, be re-instated to permanent membership. The reason for the motion was stated to be, the probable miscarriage of the notices, by which members had been caused to neglect the requirements of the Association.

Dr. WATSON, of New York, said that he considered the resolution passed at the last annual meeting in St. Louis, expelling those who had not paid their assessments or dues, an infringement of the constitution: therefore, he could see no good reason for this motion, so long as that resolution stood.

Dr. Condie rejoined, and quoted the provisions of the constitution, to show that if the payments were not made, permanent membership cannot be retained.

Dr. Watson moved to amend the motion as follows:—

*Resolved*, That no member of the Association shall be deprived of his privileges as a permanent member, by not contributing to pay the expenses of an annual meeting at which he is not present.

Considerable discussion was elicited, during which, Dr. White, of Buffalo, moved that the whole subject be referred to a committee of three. Agreed to.

The Chairman appointed Dr. White, of Buffalo, Dr. Watson, of New York, and Dr. Condie, of Philadelphia.

The list of States was now called, and each delegation reported the name of its representative in the Nominating Committee as follows:—

Maine, A. J. Fuller; New Hampshire, Silas Cummings; Vermont, Israel Hinkley; Massachusetts, C. P. Fiske; Rhode Island, Jos. Mauran; Connecticut, P. A Jewett; New York, John McCall; Pennsylvania, J. B. Biddle; New Jersey, Lewis Condict; Delaware, James W. Thompson; Maryland, Charles McGill; District of Columbia, T. Miller; Virginia, B. R. Wellford; North Carolina, O. F. Manson; South Carolina, P. C. Gaillard; Georgia, Richard D. Arnold; Alabama, P. H. Cabell; Tennessee, J. Berrien Lindsley; Kentucky, C. J. Blackburne; Ohio, R. Hills; Indiana, Joel Pennington; Illinois, J. V. Z. Blaney; Michigan, A. B. Palmer; Missouri, L. P. Perry; Iowa, J. E. Sandbourne; Wisconsin, J. B. Dousman; U. S. Navy, Thos. Dillard.

Dr. F. C. Stewart, of New York, offered the following:—

*Resolved*, That the Nominating Committee be instructed to present three names to the Association as candidates for the office of President, who shall be elected by ballot; and the candidate who shall have the smallest vote shall be withdrawn after the first ballot.

After some debate, it was moved that the resolution be laid upon the table. Agreed to.

It was moved that the Nominating Committee be instructed to recommend the place for the next annual meeting of the Convention. Agreed to.

Dr. ZINA PITCHER read an invitation from the medical profession of Detroit, to the Association, to meet next year in that city. Referred to the committee, which then withdrew for conference.

Dr. BRAINARD, of Chicago, on behalf of the medical profession of Illinois, extended an invitation to the Association to hold the next meeting in that city. He wanted the claims of Chicago remembered among the cities of the West. The invitation was referred to the Committee on Nominations.

An invitation to meet at Nashville, Tenn., was referred to the same committee.

Dr. F. A. RAMSEY, of Tennessee, moved that so much of the President's address as relates to the place of holding the meetings of the Association, be referred to the Committee on Nominations. Agreed to.

Dr. D. D. THOMPSON, of Louisville, moved a suspension of rules for the purpose of taking up that amendment to the constitution which relates to the time, in the session, at which election for officers shall take place. He stated the object of the amendment to be, to afford them sufficient time between their election and entrance upon duty, for becoming acquainted with parliamentary usages. The motion was not carried.

The next business in order was the reading of the annual reports of the Standing Committees.

The Committee on Prize Essays reported through Dr. RENE LA ROCHE. The committee had received six essays in competition for the prize offered by the Association. But, although these essays evinced much ability and extensive learning, but one was decided to possess those qualities which deserved the award of the prize. It was entitled *Statistics of Placenta Prævia.* The name of the author was announced as Dr. JAMES D. TRASK, of White Plains, Westchester county, New York. Referred to the Committee on Publication.

Dr. THOMAS REYBURN, Chairman of the Committee on the Epidemics of Missouri, Illinois, Iowa, and Wisconsin, read an abstract of the report submitted, which was, on motion, referred to the Committee on Publication.

At this stage of the proceedings, Dr. White, on behalf of the committee to whom was referred the resolution and amendments respecting permanent membership, submitted a report, recommending the adoption of the following resolutions:—

*Resolved*, That no permanent member who is not present at an annual meeting of this Association, shall be required to pay the usual assessment; but no such permanent member shall be entitled to receive a copy of the printed proceedings of the meeting, unless by paying a sum equal to that assessed upon those who were present at such meeting; and that all the names of permanent members that have been left off the published list, be re-inserted in the next volume of Transactions.

*Resolved*, That no assessment whatever shall be made against members by invitation, but that they also be entitled to a copy of the printed Transactions by paying the sum assessed upon delegates in attendance.

The report of the committee was accepted and the resolutions adopted.

Dr. Sanford B. Hunt, of Buffalo, stated that he had prepared his report on the Hygrometrical State of the Atmosphere in various localities, and its influence on Health, but it would not admit of being embodied in an abstract; its reading would consume, perhaps an hour, which he would do, if the Association so ordered; or he would furnish the manuscript to the Committee on Publication.

A motion was made to accept and refer the report to that committee, whereupon an animated discussion arose as to the propriety of allowing any report from standing committees to pass into the hands of the Publishing Committee, without first being read in abstract or in full, before the Association. The hour for adjournment having arrived, a motion to lay the subject on the table prevailed.

On motion, adjourned.

At 4 o'clock, p. m., the members visited, according to invitation, the Pennsylvania Hospital for the Insane, and in the evening they were received at the houses of Drs. Hodge, Norris, and Bache.

### MORNING SESSION.

Wednesday, May 2, 1855.

The Association re-assembled at 9 o'clock, A. M. The minutes of the preceding day were read and approved.

Dr. Jno. L. Atlee, of Lancaster, Pa., asked and received permission to make a statement from the committee appointed by the annual meeting, held at Richmond, to procure a suitable stone for the Association to contribute to the Washington Monument. An assessment of $1 had been made, upon each member, to purchase the stone and pay for the sculpture. The latter was executed by a young man named John Augustus Beck. The design was suggested by the late Dr. Pierson, of Salem, Mass., but before he could consummate the plan, the fearful Norwalk calamity swept him from our midst. The drawing was made by his daughter, Miss Abby L. Pierson. The design, cut in alto relievo, on the face of a slab of Vermont marble, is a representation of Hippocrates, when refusing the bribes offered by the King of Persia to visit the Court of that prince, and give medical aid to the plague-ridden subjects of his empire—he indignantly exclaims: "Tell your master that I am rich enough; that honor will not allow me to succor the enemies of Greece." Eminent artists, who have examined the stone, consider that no sculptor in America could have performed the work so well. Mr. Beck has been encouraged to pursue his study in Italy, and it is probable that he will rank among the finest sculptors of his age.

Dr. Atlee appealed to the members to come forward and make up a handsome compensation for the young artist.

A resolution was offered, to the effect that no member should speak unless his name and residence were announced. Adopted.

The Chairman of the Committee on Nominations recommended the following officers for the ensuing year:—

*President.*—George B. Wood, of Pennsylvania.

*Vice Presidents.*—Wm. M. Boling, Alabama; Daniel Tilden, Ohio; D. Humphrey Storer, Massachusetts; Grafton Tyler, District of Columbia.

*Secretaries.*—Francis West, Pennsylvania; R. C. Foster, Tennessee.

*Treasurer.*—Caspar Wister, Pennsylvania.

*Committee on Publications.*—Francis G. Smith, Pennsylvania, Chairman; Francis West, Pennsylvania; R. C. Foster, Tennessee; Samuel L. Hollingsworth, Pennsylvania; H. S. Askew, Delaware; Samuel Lewis, Pennsylvania.

The Committee also recommended Nashville, Tennessee, (but not unanimously,) as the next place of holding the annual meeting.

The officers recommended, were, on motion, approved by the Association.

The newly elected officers were conducted to their seats, by an appointed committee of five—Drs. J. M. Smith of New York, Homans of Boston, Blackburne of Kentucky, Rouse of Illinois, Frost of South Carolina.

Dr. Wood, on taking the chair, said he was deeply sensible of the honor conferred by the appointment, and none the less from an impression that it was probably an exhibition for the place where the Association was holding its meeting. Personally, he had a deep sympathy with the purposes for the advancement of which the Medical Association was established. He had devoted his past life to the advancement of these objects, and he would devote to the same the little that might remain. He was unaccustomed to presiding over such large assemblies, but he would endeavor to justify the appointment.

On motion, the thanks of the Association were tendered to the retiring President, Dr. Charles A. Pope, of Missouri, for the able and impartial manner in which he had presided over its meetings.

The second part of the Committee's report, advising Nashville as the place of holding the next Convention, was taken under consideration.

A member moved that the next meeting be held at Washington, District of Columbia.

Dr. Palmer offered an amendment that Detroit be substituted for Washington.

A member said that it would be impossible for the members of the Association to be accommodated in Washington, during a long session of Congress.

A member said that Nashville could be reached by railroad from the South, also, by most splendid packets, that ply on the Onio to the Cumberland river.

The motion relating to Washington was laid on the table.

Dr. Palmer, of Chicago, urged that the Association hold its next meeting at Detroit, and moved, in lieu of the report of the Committee, "that the next meeting be held at Detroit."

A motion was made that the whole subject be referred to a committee of five. Laid on the table.

The vote was taken on the motion of Dr. Palmer, "that the next meeting be held at Detroit," and it was agreed to by a large majority.

Dr. Foster, the newly elected Secretary, tendered his resignation, which was accepted.

Dr. Brodie, of Michigan, was nominated for the office, and elected by acclamation.

Dr. Hunt, of Buffalo, read an abstract of his report on the Hygrometrical State of the Atmosphere in various localities, and its influence on Health.

During the reading of this valuable paper, a motion was made and unanimously adopted, "that the desultory conversation of members be done down stairs."

Dr. Hunt's report was accepted, and referred to the Committee on Publication.

A resolution authorizing a committee to be appointed by the chair, for the purpose of obtaining commutation tickets to members over the various railroads at the time this Association was in session, was agreed to.

Dr. Frank H. Hamilton, of Buffalo, New York, read an abstract of his report "On the frequency of Deformities in Fractures."

The Doctor enjoined upon the members of the Association to carefully consider some mode of arresting the prosecutions for mal-practice, which so frequently occur in our country. He had been informed this morning, that in a district not over forty miles from this city, within a few years past, not less that twelve prosecutions had been made against members of the profession. There was no artisan but the Surgeon who was held accountable for a mere failure, and why was it that this should be? Was it the work of jealous and designing men who are in our practice, who falsely show up the failures of their brethren, and at the same time conceal their own faults? He much feared that a few of such men were among us, but was proud to repeat the remarks of an eminent lawyer, who had said of the profession "that as a whole none stands by itself so well and nobly."

Is it the fault of the lawyers? Young lawyers with neither money nor morals might have something to do with encouraging prosecutions: Joshua Spencer, an eminent lawyer, said that he had acted as counsel in many cases, but had never instituted a prosecution against any member of the medical profession; nor did he know of any member of the bar who occupied a high position that did; he had ever looked upon such prosecutions as *persecutions.*

The members of the profession should interrogate themselves as to the cause of these prosecutions and freely admit to the world the difficulties and imperfections attending Surgery, which had too long been kept concealed. We have heard members of the profession declare they could mend a fractured femur without shortening the limb; this may be an impossibility, and it is disgraceful to make such assertions, because they may not be true, and it is calculated, not only to degrade, but subject us to these prosecutions. Even that city of medical science, Philadelphia, had not produced a book which could instruct physicians how to unite a fractured femur in all cases, without shortening the limb.

The abstract and remarks of Dr. Hamilton excited great interest; on motion the report was accepted and referred to the Publishing Committee.

Dr. Charles Hooker, of New Haven, read an abstract of his report on "Diet for the Sick," which lays down laws for the government of diet under various diseases, and specifies the particular articles which may be given with benefit.

The Association here took a recess for the purpose of proceeding to Independence Hall—the members forming in line and marching in procession.

### Reception at Independence Hall.

On their assembling in the east room of this venerable and venerated Hall—the room in which was signed the document which proclaimed us to the world as a free and independent nation—the Chairman of the Committee of Arrangements, Dr. Isaac Hays, introduced the Association to the Mayor of the city in a few appropriate remarks, to which his Honor responded as follows:—

*Mr. Chairman of the Committee of Arrangements:*—I thank you, in the name of the community which I have the honor to represent, for your eloquent introduction of our friends to the authorities of the city, and to this the Hall of Independence.

*Gentlemen of the American Medical Association:* I am proud of the privilege of extending to you, in the name of the government and of the people of Philadelphia, a most cordial welcome.

I bid you welcome to our city—a city which, deriving a cherished distinction from the profession which you adorn, is eager, now and ever, to requite it, in her tribute of respect for its professors. I welcome you to our people, whose intercourse, for many a year, with you or your brethren, has inspired a feeling which, reserved as we are sometimes said to be, will, I doubt not, burst into earnest and unambiguous expression, before you leave us.

I welcome you, gentlemen, to this Hall, but not as strangers or the sons of strangers—for it is your own. As the temple and territory of Delphos, in the wildest domestic perturbations of Greece, afforded one sacred area over which the cloud of discord never gathered, one altar whose worship was never invaded, this spot, consecrated to our common American glory, knows no lines of latitude, and belongs, in truth, no more to us, whose peculiar privilege it is to inherit its guardianship, than to our brothers—*to you.* In coming hither, therefore, you *come home.* These precincts have been hallowed, for all time, by the heroic virtues of your and our fathers. This is the fountain from which the living waters of American liberty were first drawn, and it is therefore most sacred—(wo to the generation in which it ceases to be sacred!)—but, like the well of the Patriarch, all the tribes of Liberty's Israel own here an equal right, and owe here an equal homage.

In no sense, then, can I greet you as strangers—for yours are names familiar to every American proud of the science of his country; and those who are united, by this Association, in a cause so lofty as that eloquently characterized by your Chairman, may not only claim the universal and acknowledged privileges of the Republic of minds, but the rights of a nearer and a dearer character, the Brotherhood of beneficence—the kindred claims of noble hearts, knit in the highest and holiest of human aspirations. In this spirit, with the most fervent and fraternal sentiments of respect and regard, I greet and welcome you.

You are right, Mr. Chairman, in claiming, amid the associations which hallow these precincts, a peculiar privilege for your profession—a profession which not only sprinkled, with the earliest sacrificial blood of the Revolution, the highest altar upon which Valor vowed and dedicated our country to freedom—I refer as you have referred, to Dr. Warren and Bunker Hill—but which in every struggle for the enlargement and enlightenment of human destinies, has been eminently distinguished for courage, zeal, and fidelity to the rights of man. You have, therefore, a peculiar right to claim kindred here, and have that claim allowed; and within these walls, which witnessed the zeal of Rush, it would be a treason to virtue to forget, that one of the lights of your profession shed glory upon the solemn debates of this hall, and was foremost amongst those that bade yonder bell,* (preserved and devoted to the veneration of posterity), with its iron tongue, to *Proclaim Liberty throughout all the Land, to all the Inhabitants thereof.*

It is the glorious peculiarity of your profession that, while Ambition, in its ordinary and most applauded paths, plays the part of the *Destroyer*, and wins glory at the expense of human life and happiness, you and yours, with a more exalted civilization, a nobler heroism, have ever *sought to save.* Next to the highest of all human courage—if, indeed, it be merely *human*—that of the martyrs of religious Truth—the courage of the physician, whether on the battle-field or in the lazar-house, the courage of science and humanity, is most sublime, and the best entitled to the *clarum et venerabile nomen.* The vulgar courage of the warrior, under the base stimulus of passion, or the low greed of applause, can hardly be compared to the noble intrepidity of the surgeon, who gleans, in the ruthless and red-handed reaper's path, the leavings of the battle; and still less with the hero of the hospital, who encounters the grim antagonist in the horrid silence and gloom of the pestilence. Imagination can hardly embody an instance of human courage and virtue more sublime and unearthly than that of the physician, who, in the midnight of a plague-stricken city, threads the fœtid solitude of its alleys, and, entering the devoted hovel of the wretched, ministers—while only Pestilence and Misery, Death and God look on—to the perishing. I need not step from this spot to grasp the hand of many a hero who claims no laurel—many a noble philanthropist whose sacred labors, in scenes like these, have been unmarked, save by the Eye that never slumbers, and remembered only by Him who alone can reward.

To such a profession, one venerable from its antiquity, noble from the grandeur of its objects, illustrious from its achievements, and which demands every aid and energy of genius and science, of head and heart, that dignifies the race, it is not strange that, go where it may, a ready homage greets, and a ready blessing attends it. In our own city, all that is noble in patriotism, all that is exalted in science, all that is bright and beautiful in the arts which refine society, all that is lovely and cherished and holy in private life, combine to render the profession sacred and dear to us.

---

* *The Liberty Bell.*—This is the bell which was rejoicingly rung, from the steeple of the old State House, when the Declaration of Independence was originally read, in July, 1776, to the thousands assembled in the State House yard, now Independence Square. Upon this bell—cast long before the Revolution, and brought from England in the colony times—are the prophetic words of Scripture quoted—" Proclaim Liberty throughout all the Land, to all the Inhabitants thereof."

There are few living, to whom some one death in the past is not the sole event and solitary memory of the survivor's life—to him a lonely pyramid in the melancholy desert; and to such a mind and memory, *the debt of the death-bed*, where science, rendered holy by its office, ministered, though never paid, (?) is never repudiated. I never knew a good man, still less a good woman, who had not such a debt—a debt which bankrupt gratitude cherished with its holiest affections and sanctified with its devoutest memories.

In these times, when the omnipotence of associated effort is invoked for so much that is of dubious merit, it is a gratifying spectacle to behold the enlightened professors of the most exalted of all arts—men sage and grave, unselfish and unaspiring—forsaking the homes to which they are bound by the affections and the afflictions of thousands, by wealth, fame, and influence, to wander, wearily, away upon a pilgrimage of hundreds of leagues, in the cause and interests of the human family, its security, health, and happiness. For more than ten years, the representatives of your profession have thus gathered in Convention. What other body of our citizens have made a like effort—a like sacrifice? Selected from the most eminent of the profession, the delegates have been men whose years, like their virtues, were many. How difficult must have been, to them, the effort to burst through the bonds of a relying and clinging practice! How great the labor and how heavy the sacrifice! They have already visited, in this duty, the cities of every section of our wide country. How many have fallen by the wayside? How many martyrs could you not thus number in this cause? How many of the good and great of the profession have, in these benevolent pilgrimages, joined the ranks of the thousands who have sacrificed themselves, at the requisitions of duty, as recognized and enforced by your self-imposed laws—joining the dead in the effort to aid the living? The epitaph of the Spartans at Thermopylæ might well commemorate the virtues and the fate of these martyrs. But if the cost has been great, the results have been commensurate.

Of the professional advantages attained, though I know them to be invaluable, I will not presume to speak; but I may be permitted to state, as health is the most important subject of municipal provision and care, that the Transactions of the Association, which I have examined with great interest, comprise much that merits the attention, and will reward the respectful consideration, of the municipal governments of the Union.

It is natural that Philadelphia should feel, as she does feel, a profound interest in the cause of medical education in this country. She cannot, of course, forget that it was here that the first medical college was established in this country; that its merits and success extorted a reluctant transatlantic tribute of admiration; and that, progressing rapidly, but wisely, it achieved and maintained an equality with the most celebrated institutions of the Old World. As the cause of medical education has expanded, and institutions worthy of the cause and the country have sprung up, each triumph, thus attained, has been regarded here as the successful outbursting of an offshoot from the primary effort; and Philadelphia, while rejoicing in the expansion and elevation of medical education throughout the land, has almost fancied—so earnest is her interest in medical education—that she had a right to indulge a *parental* pride in all that advances that interest.

These genial feelings have been maintained, in all their early and fervid freshness, by constant intercourse with all sections of our country. The ingenuous and gallant youths that have come hither for medical instruction have, in their unstudied intercourse, exhibited the character of their respective States in a light so generous and exalted, as to win our affections, not only for themselves, but for the communities and States which could exult in them as their own. Winter after winter, we have had many hundreds of these noble young spirits among us. And let me remark that, rigorous as I am said to be in the administration of the law, I have yet to know the first occasion to rebuke, much less to punish, a medical student. We have found them as gentle and decorous in their deportment, as they are exalted in their aspirations; and had Philadelphia—eminently catholic in her affection for her sister communities—needed a lesson of love and loyalty, these high-hearted missionaries would have taught it. This interchance of sympathies has endured for the third of a century—may it last forever! The youths—youths no longer—who formerly bore those sentiments to the remote sections of our republic, stand before me now as the revered sages and ornaments of their profession, meeting here the evidences of a reputation which had preceded them, and has long been cherished by us. And who can tell what have been the results of this kindly interchange of kindly feelings? It has doubtless been felt in every commercial, social, and political relation of life, correcting the prejudices, harmonizing the discords, and subduing the dangers of our comman country.

We realize these facts. We recognize in the members of an enlightened profession like yours, so many patriots and philanthropists engaged in the great and general interests of the human race; and, apart from the mere scientific acquisitions of your annual meetings, we perceive in them results auspicious to all that we cherish, all that is kindly, forbearing, and conservative, between man and man, party and party, state and state, section and section; and, so regarding them, we hail and greet you with a welcome as sincere and cordial as the heart can conceive, or the tongue can utter.

### AFTERNOON SESSION.

At 1 o'clock, P. M., the members having returned from Independence Hall, the President called the Association to order.

On motion, the thanks of the Association were tendered to the Mayor of Philadelphia for the very cordial manner in which he had received the Medical Association at Independence Hall, and that a copy of his speech be requested for publication in connection with the proceedings of the Association.

The rules were suspended, when Dr. THOMPSON, of Delaware, offered the following preamble and resolutions:—

*Whereas*, Few subjects of greater interest and importance could be presented to the consideration of the American Medical Association, now representing most of the States and Territories of the Union, than the attainment of a correct medical topography of each, with a history of its prevailing fevers, and the most successful treatment of the same; therefore, be it

*Resolved*, That with this view and conviction, that this Association now appoint a special committee from each State and Territory represented, of —— members, whose duty it shall be to report upon its medical topography, epidemic fevers, and the most successful treatment thereof, and that the same shall continue to hold their office for three years.

*Resolved*, That in the appointment of gentlemen of education and experience in the affairs of their own State, we have the best guarantee that the important objects we seek will be most satisfactorily accomplished; and the profession, as well as the public interest, will thereby be better served.

*Resolved*, That the committees heretofore appointed by this Association, at its session in Charleston, for a similar object, be, and the same are hereby discharged.

Dr. ASKEW, of Delaware, seconded the resolutions, and moved that they be laid on the table, and made the special order for Thursday at 10 o'clock.

The motion was agreed to.

The Committee on Publication submitted a report, stating that the seventh volume of the proceedings of the Association was issued last November, 1,000 copies being published at an expense of $1,806 42; 781 copies have been sold or furnished to members of the Association; 35 were given to editors of medical journals, and 184 remain on hand. The resolutions, appended to the report were adopted.

Dr. BIDDLE, of Pa., offered the following resolution, which was adopted:—

*Resolved*, That the thanks of the Association are eminently due to the Committee on Publication, for the faithful and highly satisfactory manner in which their arduous and responsible duties have been discharged.

The Treasurer, Dr. ISAAC WOOD, presented his report, showing that the sum of $2,101 04 was expended, during the past year, for the purposes of printing, binding, engravings, prize essays, &c.

The balance received from Dr. D. F. CONDIE, the former Treasurer, was $293 99, while the assessments and sale of Transactions amounted to $2,722 31. There was also $200 received from Drs. GEORGE B. WOOD and DANIEL BRAINARD on account of the prize fund, thus leaving a balance on hand of $1,115 26.

Dr. CONDIE read a communication exculpating the former Committee on Publication from certain charges made against it at the meeting of 1854, in regard to the publication of the proceedings; and a charge made against Dr. MEIGS, of this city, of making profit by publishing the material of the Association, as his own work. The charges against the committee were, that they had delayed the publication of the last volume of the proceedings, and had excluded several papers that were presented at the meeting of the Association. Both these charges were disproved, and Dr. MEIGS was exonerated from the charge made against him. Dr. CONDIE asked that the communication be entered upon the journal.

A resolution was offered by Dr. STEWART, of New York, thanking the former Committee on Publication for the faithful performance of their arduous duties, and expressing the satisfaction of the Association therewith.

Dr. CONDIE stated that the Committee on Publication cared but little for the charges made against them; but they desired to do justice to a man who stood very high in reputation in Philadelphia, and who felt himself aggrieved by slanderous accusation. He withdrew the request that his communication be entered upon the minutes.

The resolution of thanks was then withdrawn, as Dr. CONDIE did not think it necessary.

Dr. WATSON offered a resolution providing for the appropriation of $1,000 to pay for the stone for the Washington Monument, which was adopted.

Dr. W. I. C. DUHAMEL, of Washington City, offered the following preamble and resolution:—

*Whereas*, The cause of humanity, and a due regard to public health, imperatively demand the speedy enactment of more stringent quarantine laws to prevent the spread of infectious diseases throughout the country; *And Whereas*, Distinguished legislators have co-operated with the medical profession in bringing this important subject to the attention of Congress; therefore

*Resolved*, That warmly approving of the bill introduced at the last session of Congress, and yet pending, to prevent sickness and mortality on shipboard, we cordially tender the honorable senators of the Special Committee, and others who so ably and earnestly advocated the measure, this expression of our sincere thanks for their unwearied exertions in behalf of the bill recommended by this Association, and yet indulge in the gratifying hope that their efforts in the cause of public welfare and suffering humanity will be crowned with success.

Dr. MAURAN, of Providence, Rhode Island, seconded the above, and they were unanimously adopted.

The Secretary read a special report from Dr. WM. H. BYFORD, of Evansville, Indiana, upon the Pathology and Treatment of Scrofula. This paper gives an account of the nature of the disease its varieties, causes, effects, and treatment. Referred to the Committee on Publications.

Dr. N. S. DAVIS, of Chicago, presented a report on the "Nutritive Qualities of Milk, and the influence produced thereon by Pregnancy and Menstruation in the Human Female, and by Pregnancy in the Cow; and, also, on the question whether there is not some mode by which the nutritive constituents of milk can be preserved in their purity and sweetness, and furnished to the inhabitants of cities in such quantities as to supersede the present defective and often unwholesome modes of supply."

The report says:—

When railroads were opened into the interior of the country, it was said that milk would be furnished to residents of cities in the purity that it was found on farms; but a sufficient time had elapsed to demonstrate that such is not the case. The conveyance of the milk from the farm to the cars, the transit on the railway, and the time lost in its delivery throughout the city, it was clearly shown, had the effect of making it unfit for the nourishment of a child. During the past half century experiments had been made with a view of preserving milk in its pure state, yet it was but recently that a discovery had been made by a gentleman in New York, which was to evaporate the water, and mix with white sugar, which rendered it what is termed, solidified milk. In his practice he had used this improved milk for the nourishment of infants with the most gratifying results, and after having kept it for three months; and he knew of its having been kept twelve months without any injury to its qualities.

The report was referred to the Committee on Publication, and the meeting adjourned.

In the course of the day's proceedings, the Chairman announced that visiting invitations had been extended to the delegation from Prof. Hart, of the Central High School; from the Board of Health, to visit the City Hospital and the Lazaretto, which were accepted with thanks. At 4 o'clock, the Association, to the number of five hundred, proceeded in coaches to the reception at Girard College, after which they proceeded to Fairmount.

In the evening they were entertained at the residences of Drs. Wood, A. Stillé, and Paul.

---

### MORNING SESSION.

THURSDAY, May 3, 1855.

At 9 o'clock, the attendance was comparatively slim, but the members continued dropping in, and the spacious Hall was soon filled up.

The Chair was taken by the President, Dr. WOOD, who called the Association to order.

The Secretary, Dr. WEST, read the minutes of yesterday's meeting, which were approved.

The chair said that there was no prescribed way of appointing the Committees on Prize Essays, Arrangements, &c.; it had been usual, however, for the Convention to refer the appointment of these committee, on this occasion. The motion was agreed to.

Dr. ISAAC HAYS, from the Committee of Arrangements, announced that five hundred and twenty delegates were now enrolled.

Dr. HAYS presented an invitation from Dr. Ducachet, of St. Stephen's Church, to visit the church and see the Byrd monument, by Steinhauser. He stated that 12 o'clock at noon, on this day, had been fixed for this visit. The invitation was accepted.

A letter was read from Dr. Reyburn, of Missouri, suggesting that the large district in Missouri be divided into two parts. The district is seven hundred miles north and south, and four hundred miles east and west. The duties in so large a district as this, cannot be easily fulfilled by one chairman. By dividing the district, the reports will be more satisfactory than at the present time. The Doctor tendered his resignation.

Dr. WATSON moved the acceptance of the resignation, and the reference of the other portion of the communication to the Committee on Nominations; which was afterwards withdrawn.

Dr. ASKEW renewed the motion, and it was adopted.

A letter was read from Dr. E. B. HASKINS, of Clarksville, Tenn., chairman of the Committee on Microscopical investigations of Malignant Tumors, asking to be excused from making a report, inasmuch as he had not the necessary apparatus for ascertaining the facts incident to the subject.

The request of the gentleman was granted.

A report was received from the Committee on Publications, adverse to the publication in the Transactions of the reports of Special Committees. Referred to a Special Committee of five.

Dr. FRANK H. HAMILTON, of Buffalo, New York, made some additional remarks on the subject of fractured clavicle. The Doctor expressed an earnest hope that the Managers of the Pennsylvania Hospital would be more exact in making up their reports of the statistics of fractured clavicle, so that we may all be able to judge of the merit of the instrument, which has been used in such cases in that Institution, for thirty years. He had known a surgeon to be mulcted in heavy damages for using that instrument in a case of fractured clavicle, because he could not accomplish all that he supposed he could by using it. He did not wish to speak harshly, but at the same time he called again upon the Managers of that Institution to be more exact in their statistics on the subject of fractured clavicle, so that the medical faculty may be prevented being a grand Insurance Company for the whole world.

Dr. THOMPSON, of Delaware, called up his resolutions, offered yesterday, in reference to the appointment of committees on the Medical Topography of the country.

Dr. J. L. ATLEE, of Lancaster, said that he noticed Chief Justice

Lewis, of Pennsylvania, on the floor of the house—he moved that he be invited to a seat on the platform. The motion was unanimously agreed to.

The Chief Justice was (amid much applause) escorted to the platform and seated on the right of the President.

Dr. ASKEW, of Delaware, offered the following as an appendage to the resolutions of Dr. Thompson:—

*Resolved*, That all reports on the Medical Topography and prevailing diseases of States, shall, to entitle them to be received by this Association and published in the proceedings, be first approved by the Medical Societies of the State or Territory where such Societies exist, and to which State or Territory such report refers.

Dr. PALMER, of Michigan, advocated the resolutions, and said that, while he was conscious of the difficulties in the way, he thought that great good would result from the appointment of the committees.

Dr. J. G. ORTON, of Binghampton, N. Y., offered the following resolutions:—

*Resolved*, That each County Medical Society (or in parts of the country where such have not been established, any duly organized Medical Association) be requested to amend its Constitution by attaching thereunto the following article: "It shall be the duty of each member of this Society to keep a faithful record of the diseases which may fall under his observation during each month according to the classification adopted by the American Medical Association in May, 1847, stating the age and sex, occupation and nativity of the patient, the average duration of the disease, and finally their recovery or death, and to report the same in writing to the Secretary on or before the first day of February of each year, who shall transmit a digest thereof to the State Medical Society, and also to the appropriate Committee appointed by the American Medical Association for its reception."

*Resolved*, That each incorporated Hospital, Infirmary and Asylum, be invited to furnish a copy of their annual reports for the use of the Committee of their respective States.

*Resolved*, That the State Committee appointed by this Association to report on the prevailing diseases of their respective localities, shall receive and arrange a digest of the reports transmitted to them by the Secretaries of the various County Societies, and to report the same at the annual meeting of this Association.

*Resolved*, That the 1st day of January be the time fixed at which the object of these resolutions shall be carried into effect, and that the several County Societies and Associations, be requested to amend their Constitutions as heretofore recommended, at as early a date as practicable, and to report to the State Committee their willingness or unwillingness to acquiesce in the request of this Association.

On motion, the resolutions were referred to the Committee on Nominations.

Dr. THOMPSON, of Delaware, expressed a hope that the Committee would go into immediate session, so that a report might be made as soon as possible.

Letters from Drs. SUTTON and FENNER, on the subject of epidemics, were referred to the Committee on Nominations.

Dr. CONDIE, of Philadelphia, submitted a voluminous report upon the subject of Tubercular Disease, accompanied by a vast array of facts, the gathering of about three years. He stated that the report would make at least 500 printed pages. The opinions advanced, he said, were "very heterodox."

On motion, Dr. CONDIE was permitted to publish the report in any manner he pleased, without reference to any obligations he may feel due to the Association.

Dr. BRAINARD, from the Special Committee on the constitutional and local treatment of Carcinoma, was continued another year.

Dr. HORACE GREEN, from the Special Committee on the use and effects of applications of Nitrate of Silver to the Throat, either in local or general disease, was also continued another year.

A member from the Committee on Dysentery submitted a lengthy report upon the nature and treatment of that disease. Referred to the Committee on Publications.

The hour of recess having arrived, the members took advantage of invitations to visit the Byrd Monument, in St. Stephen's Church, the Academy of Fine Arts, the Academy of Natural Sciences, and the Central High School.

---

### AFTERNOON SESSION.

The Association re-assembled at 1 o'clock.

Dr. MUSSEY, of Ohio, read an interesting report upon the Use of Alcohol in Health and Disease. It abounded in facts illustrating the effects of alcohol upon the human system, and evinced extensive reading, and a laborious collation of medical testimony. Referred to the Committee on Publications.

Dr. Hays presented an invitation to the Association to visit the Pennsylvania Institution for Idiotic and Feeble-Minded Children, this afternoon. Coaches will be provided to take the members to Germantown. The invitation was accepted.

The Committe on Nominations to which was referred so much of the President's address as relates to the next place of meeting, recommended that no change be made in the place of holding the next annual meeting—which will consequently be held at Detroit.

A letter was read from Dr. R. J. Breckenridge, of Kentucky, chairman of the Committee of Medical Literature, announcing that his report was ready, but that he was not able to present it himself, and asking its reference to the Committee on Publications.

Dr. Condie moved, that when received, the report be so referred.

A communication was read from Dr. W. H. Anderson, of Mobile, chairman of the Committee on Medical Education, setting forth that his duties requiring an extensive foreign and domestic correspondence, his report was incomplete, but would be ready in season for publication in the Transactions.

A motion was made that the report, when received, be referred to the Committee on Publications.

Dr. Davis, of Illinois, hoped that the paper would not be referred without first being read. He was opposed to the principle of referring papers to the Committee on Publications, until the Association had had an opportunity of judging of their merits.

Dr. Merritt moved that the vote adopting the report of the Nominating Committee upon the subject of the appointment of committees be reconsidered, for the purpose of continuing two of the old committees.

This motion was suspended to admit the report of the Nominating Committee upon the resolutions in regard to the Medical Topography of the States and Territories.

The vote respecting the adoption of the report of the Committee on Nominations, in regard to the appointment of committees, was reconsidered, and Dr. Anderson was continued as chairman of the Committee on Medical Education, in lieu of Dr. J. B. Lindsley.

A resolution in regard to the registry of births and deaths was referred to the Nominating Committee.

On motion, the Association adjourned, it being 3 o'clock.

At 4 o'clock the members of the Association entered coaches and proceeded to the City Hospital, Blockley. They were received by the President of the Board of Guardians, Mr. F. M. Adams, and the Chief Resident Physician, Dr. A. B. Campbell, and conducted through the Hospital, and the Almshouse. In the evening, the members of the Association were received at the residences of Drs. Samuel Jackson, J. Pancoast, Henry Hartshorne, and Mr. Isaac Lea.

---

### MORNING SESSION.

Friday, May 4, 1855.

The Association assembled at nine o'clock, pursuant to adjournment, Dr. Wood in the chair.

The Secretary read the minutes of yesterday's meeting, which were approved.

Dr. Hays stated that the whole number of names registered now, amounted to five hundred and twenty-three.

Dr. Atlee offered a resolution that the report of Dr. Breckenridge, chairman of the Committee on Medical Literature, be referred to a committee of three for their approval, and then be handed to the Committee on Publications.

A member moved it go to the Committee on Publication direct, without any previous review.

Dr. Condie hoped that a report on such an important subject would not go to the Committee without having been read before the Association. He hoped the Committee would be continued, so that the chairman could be present next year, and read it.

The motion of Dr. Atlee was lost.

The motion to continue the Committee was agreed to.

Dr. Hayward offered a resolution expressing the thanks of the Association to all who had, by attention and munificent hospitality contributed to the enjoyment of the delegation. Unanimously adopted.

The committee appointed at St. Louis, to whom had been referred the paper of Dr. Phelps, entitled "Religion an Element of Medicine, or the duties and obligations of the Profession," reported through Dr. J. L. Atlee, who moved that it is inexpedient to publish Dr. P's. paper in connection with the transactions of the Association.

The report was adopted as the sense of the meeting, and the resolution agreed to.

A member offered a resolution returning thanks to the Directors of the Academy of Fine Arts, for their liberal offer to take charge of the stone for the Washington Monument, and transport it to Washington. Agreed to unanimously.

Dr. N. S. Davis, of Illinois, moved the following preamble and resolutions, which were referred to the Committee of Arrangements with instructions to report on the same at the commencement of the next annual meeting:—

*Whereas*, The present mode of conducting the annual meetings of the Association affords but little opportunity for the discussion of strictly scientific questions and papers; *And Whereas*, this has been regarded as a serious defect in the operation of our organization, impairing its scientific character; therefore

*Resolved*, That the daily sessions of the Association during each annual meeting be divided into two parts, the first to terminate at an hour not later than 12½ o'clock, P. M., of each day, and to be devoted as heretofore, to the general business of the Association. The second part consisting of all the time which it is deemed advisable to remain in session each day, after 12½ o'clock, P. M., to take the character of a scientific session, and to be devoted exclusively to the discussion of questions relating to the science and art of medicine.

*Resolved*, That the Association, in its capacity of a *Scientific Section*, having no power to act on any subject of a *scientific* character, may continue in session, whenever thought desirable, a longer period than in its more general capacity.

Dr. A. J. Semmes, of Washington, D. C., offered the following which was adopted:—

*Resolved*, That a committee of three be appointed to report to the Association, at its next annual meeting, what measures should be adopted to remedy the evils existing in the present methods of holding coroners' inquests by incompetent persons, by which the lives and liberties of the innocent may be jeopardized, and the ends of justice frustrated.

Dr. SEMMES, of Washington, Dr. HYLE, of Wilmington, and Dr. CONDIE, of Philadelphia, were appointed.

Dr. J. L. ATLEE offered the following resolutions, which were intended to counteract a recent recommendation of the Legislature of Michigan:—

*Resolved*, That to secure efficient teaching in medical schools, where a prime object is to enforce practical precepts, a large degree of union and harmony must exist among the teachers, and confidence be reposed in them on the part of their pupils.

*Resolved*, That any such unnatural union as the mingling of an exclusive system, such as Homœopathy, with Scientific Medicine, in a school, setting aside all questions of its untruthfulness, can not fail, by the destruction of union and confidence, and the production of confusion and disorder, unsettling and distracting the minds of the learners, to so far impair the usefulness of teaching, as to render any school adopting such a policy unworthy the support of the profession.

Dr. A. B. PALMER, of the University of Michigan, in seconding the resolutions, desired permission to offer a few remarks. After briefly stating some of the peculiar features of the incorporation of the University, he went on to say that the Institution is under the management of a Board of Regents elected by the people, by whom the professors in the various departments of science and art are appointed. The Legislature has nothing to do with the appointment of professors. During the session of the Legislature the past winter, the admirers of homœopathy had made special effort, and had succeeded in obtaining a *recommendatory* action on their part, in reference to the establishment of a chair of homœopathy in the University. It had been represented to them, that a large part of the people of Michigan favored that peculiar mode of practice, and that it was but fair that its principles be inculcated in the State University.

The spirit of this recommendation is, and has been resisted, both by the Regents, and by the Faculty and Students of the medical department of the University.

Dr. PALMER wanted an expression of opinion by the Association on this matter. Legislatures should know that Homœopathy and Scientific Medicine should not, and cannot be united in one school —they are incompatible, and neither he or any of his colleagues could, or would continue in any school where such an absurdity was attempted.

Some of the medical journals have given an erroneous impression in regard to this action of the Legislature. The faculty are going to act energetically in this matter, and they ask the Association to co-operate with them in instituting a thorough and systematic inquiry, both in this country and in Europe, into the pretensions and practice of this delusion and imposture, that the minds of the people may be enlightened on the subject. As the friends of Homœopathy in Michigan have raised the issue, the faculty of the University wish to meet it. Circulars of inquiry will soon be sent out, and the faculty hope that they will be promptly responded to.

The Doctor repeated the assurance that on no account would the faculty tolerate a Chair of Homœopathy in the University, but that, to a man, they would resign their positions rather than submit to so absurd and humiliating an appointment.

The doctor's remarks were received with evident satisfaction by the members, as was shown by the frequent rounds of applause that greeted him.

Dr. Clendenin, of Ohio, offered the following:—

*Resolved*, That no State or Local Society shall be hereafter entitled to representation in this Association that has not adopted its Code of Ethics.

*Resolved*, That no State or Local Society that has intentionally violated, or discarded any article or clause in the Code of Ethics, shall longer be entitled to representation in this body.

A motion to lay the above on the table was lost.

Dr. Miltenberger, of Baltimore, offered an amendment, which was amended by Dr. J. L. Atlee to read as follows:—

*Whereas*, It has been brought before the notice of the American Medical Association that the State Medical Society of Ohio has violated, at their last meeting, Sec. 4, Art. 1, Chap. 2, of its Code of Ethics; therefore be it

*Resolved*, That the Secretary of the Association be directed to inform the officers of that Society that, unless such action be rescinded, they cannot be hereafter represented in this Association.

These were added to the resolutions of Dr. Clendenin, and adopted unanimously; the delegates from the Society in question, being forward in support of them.

Dr. Alfred Stille offered the following resolutions:—

*Resolved*, That a Special Committee of five be appointed to report, at the next meeting of the Association, on the following question: Might not the present system of repeating the same lectures to the same classes, during two successive terms, be usefully modified by extending the lectures of each chair over two sessions, so as to embrace a systematic and complete discussion of each of the following subjects:—

1. Special, Regional, and General Anatomy, including illustrative references to Morbid Anatomy.
2. Inorganic, Organic, and Pharmaceutical Chemistry, and Toxicology.
3. General and Human Physiology; Hygiene; Medical Jurisprudence.
4. Medical Botany; Materia Medica; General Therapeutics.
5. General Pathology; Morbid Anatomy (Systematic); Practice of Medicine.
6. General Surgical Pathology, or Institutes of Surgery; Mechanical, Operative, and Medicinal Surgery.
7. Obstetrics; Diseases of Women; Diseases of Children.
8. Hospital Clinical Medicine and Surgery.

*Resolved*, That the Committee, at an early day, address the several Medical Colleges, in regard to the proposed plan of instruction, requesting from them an official expression of opinion upon its merits and feasibility.

The Chair appointed the following gentlemen as the Committee, to whom the subject is referred:—

Dr. Alfred Stillé, Philadelphia, Chairman, Prof. Samuel Jackson, Philadelphia, Dr. John Bell, Philadelphia, Dr. John Watson, New York, Dr. J. L. Cabell, Charlottsville, Va.

Dr. Corson, of New York, read a volunteer report on the Influence of Lead on the Heart, which was referred to a special committee of three for examination.

The hour of recess now arrived, and a motion prevailed to dispense with the recess, and remain in session until final adjournment.

Dr. Thomas, of Baltimore, explained a simple method of preparing nitrate of silver for inhalation in diseases of the throat and chest. The apparatus was placed on the table, and, when put in motion, the nitrate of silver was thrown off in an impalpable powder. The subject was referred to a special committee of three.

Dr. Atlee called up a resolution, offered at the last meeting of the Association, directing the especial attention of the Committee on Epidemics to the subject of the contagiousness or non-contagiousness of cholera. The resolution was then adopted.

Dr. Davis said that the Association had returned thanks piecemeal, and he was very much afraid that some of the institutions visited had been overlooked. He moved that the Secretary be instructed to insert the names of any that had been omitted. Agreed to.

### Amendments to the Constitution.

The amendment to the Constitution, proposed by Dr. Gross at the last meeting, providing for the election of officers immediately before the adjournment of the Convention, instead of at its commencement, was called up, and, on motion of Dr. Davis, it was indefinitely postponed.

The amendment to the Constitution, providing that the Association meet on the second, instead of the first Tuesday of May, was called up, and its approval advocated by several of the New York delegates. A motion for indefinite postponement was agreed to.

Dr. Davis called up an amendment to the Constitution, offered at the last meeting, dispensing with the Nominating Committee, and then moved that it be indefinitely postponed. Agreed to.

The amendment to the Constitution, to change the title of "Committees on Epidemics," to "Committees on Prevalent Diseases," was, on motion, indefinitely postponed.

A member offered an amendment to the Constitution, by which the Recording Secretary and Treasurer of the Association are made permanent officers, and their travelling expenses directed to be paid. Laid over under the rule.

The following amendment to the Constitution was offered and laid over until the next meeting:—"Any permanent member, who shall not pay for the published Transactions for three successive years, shall be considered as having withdrawn."

An amendment was offered to the Constitution, changing the name of the Association to that of "National Medical Congress," and providing that at least one meeting in three years be held in Washington, D. C. Laid over under the rule.

An amendment to the Constitution was offered to the effect that Medical Societies which do not adopt the Code of Ethics shall not be considered "in good standing." Laid over under the rule.

Dr. L. D. SCHEETZ, of Ohio, presented the following—

*Whereas*, It has been found necessary to adopt some means of elevating the standard of education, both professional and general, among medical men, by those who are best acquainted with the sad and lamentable deficiency which prevails in this respect among a large mass of the profession, and particularly in the Western States; *And Whereas*, Efforts to this effect at home have been opposed on the ground that more was exacted by such as took an active part in the matter than is required by the "American Medical Association;" *And Whereas*, It is believed that the National Society can exert a beneficial influence over the whole country; therefore

*Resolved*, That the Constitution of this Association be so amended as to require all delegates, before being allowed a seat in said Association, to satisfy the proper authorities that the Societies which they represent, require graduation as a sine qua non to membership therein; and that no person can become a permanent member, a member by invitation, or can be received as a delegate from any other body, unless he be a graduate of some respectable school.

*Resolved*, That the editors of the various medical journals of the United States be requested to publish the foregoing, so that an interest may be awakened upon this subject, and that Societies may be prepared to comply with the above requisitions, in case they meet the approval of this Association. Laid over under the rule.

### REPORT OF COMMITTEE ON NOMINATIONS.

The Committee on Nominations reported the following committees for the present year:

*Committee on Prize Essays.*—Drs. A. B. Palmer, Samuel Denton, A. R. Perry, Abram Sager, S. H. Douglass, Corydon La Ford, E. Andrews, all of Michigan.

*Committee of Arrangements.*—Drs. Zina Pitcher, Moses Gunn, G. B. Russell, A. S. Leland, Moses Stewart, Peter Klein, and J. A. Brown, of Detroit.

*Committee on Plans of Organization for State and County Societies.*—Drs. A. B. Palmer, Michigan; N. B. Ives, Connecticut; E. B. Haskins, Tennessee; Chas. Woodward, Ohio; Josiah Crosby, New Hampshire.

*Committee on Medical Literature.*—Drs. P. C. Gaillard, South

Carolina; N. P. Monroe, Maine; James Couper, Delaware; R. Hills, Ohio; A. Coffin, South Carolina.

*Committee on Medical Education.*—Drs. W. H. Anderson, of Alabama; J. B. Flint, Kentucky; P. H. Cabell, Alabama; Geo. Hayward, Massachusetts; E. B. Smith, Missouri.

*Committee to procure Memorials of the eminent and worthy Dead.*—Drs. P. A. Jewett, Conn.; Thos. F. Betton, Pa.; C. J. Blackburne, Ky.; Wm. M. Boling, Ala.; Zina Pitcher, Mich.

*Committee on Medical Topography.*—J. C. Winston, of Bangor, Maine; Edmund R. Peaslee, of Dartmouth College, New Hampshire; Joseph Perkins, of Castleton, Vermont; Geo. C. Shattuck, of Boston, Massachusetts; Joseph Mauran, of Providence, Rhode Island; Chas. Hooker, of New Haven, Connecticut; Joseph M. Smith, of New York city; Jacob M. Gemmil, of Hollidaysburgh, Pennsylvania; Lyndon A. Smith, of Newark, New Jersey; Jas. M. Thompson, of Wilmington, Delaware; Peregrine Wroth, of Chestertown, Maryland; Thomas Miller, District of Columbia; J. F. Peebles, of Petersburgh, Virginia; O. F. Manson, North Carolina; D. O. Cain, of Charlestown, South Carolina; John F. Posey, of Savannah, Georgia; S. W. Clauton, of Warsaw, Ala.; T. O. Grafton, of Rodney, Mississippi; E. D. Fenner, of New Orleans, Louisiana; E. B. Hoskins, of Clarksville, Tennessee; W. L. Sutton, of Georgetown, Kentucky; G. Mendenhall, of Cincinnati, Ohio; Vierling Kersey, of Milton, Indiana; J. H. Beach, of Cold Water, Michigan; John Evans, of Chicago, Illinois; J. B. Alleyne, of Saint Louis, Missouri; A. S. Castleman, of Delafield, Wisconsin; E. A. Arnold, of Davenport, Iowa; J. H. Murphy, Falls of St. Anthony, Minnesota; Thos. Dillard, of Philadelphia, U. S. Navy; Clement A. Finley, U. S. Army.

*Committee on Registration of Marriages, Births, and Deaths.*—Drs. R. M. Wilson, Hartford, Connecticut, chairman; G. S. Palmer, Gardiner, Maine; Silas Gumming, Fitz William, New Hampshire; G. T. Elliott, Woodstock, Vermont; Ed. Jarvis, Dorchester, Massachusetts; Jos. Mauran, Providence, Rhode Island; Jno. H. Griscom, New York city; H. Carpenter, Lancaster, Pennsylvania; O. H. Taylor, Camden, New Jersey; Lewis P. Bush, Wilmington, Delaware; A. Snowden Piggot, Baltimore,

Maryland; David H. Tucker, Richmond, Virginia; —— Pitman, Tarboro', North Carolina; Harry Lindsly, Washington, District of Columbia; Jno. L. Dawson, Charleston, South Carolina; R. D. Arnold, Savannah, Georgia; A. Lopez, Mobile, Alabama; Jas. Jones New Orleans, Louisiana; R. C. Foster, Nashville, Tennessee; C. J. Blackburne, Covington, Kentucky; Jno. Dawson, Columbus, Ohio; Edward Murphy, New Harmony, Indiana; A. D. Stebbins, Detroit, Michigan; J. V. Z. Blaney, Chicago, Illinois; Geo. D. Wilber, Mineral Point, Wisconsin; Wm. M. McPheeters, St. Louis, Missouri; J. D. Elbert, Keosauqua, Iowa; Jno. H. Murphy, Falls of St. Anthony, Minnesota.

Mississippi and Arkansas blank.

*Special Committees.*—Dr. Lewis H. Steiner, of Washington, District of Columbia, on Strychnia—its chemical and toxicological properties.

Dr. Ashbury Evans, of Covington, Centucky, on Tracheotomy in Epilepsy.

Dr. J. Taylor Bradford, of Augusta, Kentucky, on the treatment of Cholera.

Dr. Charles Q. Chandler, of Rochester, Missouri, on Malignant Periodic Fevers.

Dr. H. A. Johnson, of Chicago, Illinois, on the Excretions as an index to the Organic Changes in the System.

Dr. Henry J. Bigelow, of Boston, Massachusetts, on Microscopical Investigation of Malignant Tumors.

Dr. E. H. Davis, of New York, on the Statistics of Calculous Diseases, and the operations therefor.

Dr. J. S. Carpenter, on the Treatment and Curability of Reducible Hernia.

Dr. A. J. Fuller, of Maine, on the Best Treatment of Cholera Infantum.

Dr. William B. Page, of Philadelphia, on Injuries of the Joints.

Dr. Wilson Jewell, of Philadelphia, on the Statistics of Mortality in the United States.

Dr. J. Knight, of New Haven Connecticut, on Endemic Fevers.

Dr. P. H. Cabell, of Alabama, on the Native Substitutes for Cinchona, indigenous to the Southern States.

Dr. James M. Newman. of Buffalo, New York, on the Sanitary Police of Cities.

Dr. L. M. Noble, of Le Roy, Illinois, on Puerperal Fever and its Communicability.

Dr. J. M. Freer, of Chicago, Illinois, on the Progress of General and Descriptive Anatomy.

Dr. J. M. Corson, of New York, on the Causes of the Impulse of the Heart, and the Agencies which Influence it in Health and Disease.

Dr. D. Meredith Reese, of New York, on the Causes of Infant Mortality in large Cities, the Source of its Increase, and the Means for its Diminution.

Dr. Mark Stephenson, of Vermont, on the Treatment best adapted to each Variety of Cataract, with the Method of Operation, Place of Election, Time, Age, &c.

Dr. J. B. Coleman, of New Jersey, on the Effect of Mercury on the Living Animal Tissues.

Dr. T. G. Richardson, of Louisville, Kentucky, on the Diversity of the Venereal Poison.

Dr. J. B. Flint, of Louisville, Kentucky, on the best mode of rendering the medical patronage of the National Government tributary to the honor and improvement of the Profession.

Dr. M. M. Latta, of Goshen, Indiana, on whether there are any means by which the growth of the Fœtus in Utero may be controlled without injury to mother or child.

Dr. Thos. Miller, of Washington, District of Columbia, on Toxicology.

Dr. E. R. Peaslee, of Hanover, New Hampshire, on Inflammation, its Pathology, and its relation to the Reparative Process.

Dr. D. D. Thompson, of Louisville, Kentucky, on the Remedial Effects of Chloroform.

Dr. William Clendennin, of Cincinnati, Ohio, on Epidemic Erysipelas.

Dr. C. G. Comegys, of Cincinnati, Ohio, on the State of the Urine in Tubercular Disease.

Adjourned.

