# Correction

**Published:** 1855-07

**Authors:** 


### CORRECTION.

At the request of Prof. Frank. Hamilton, of Buffalo, N. Y., we cheerfully copy the remarks he made at the late meeting of the A. M. A. from the Medical Journal, as they are more complete, and differ in some material points from those contained in our June number:

"Dr. Hamilton said he had a word to say which did not belong to the report. Prosecutions for malpractice have become so frequent that surgeons were alarmed, and not a few were abandoning the profession, or refusing altogether to undertake the treatment of grave surgical accidents, and especially of fractures. So frequent were these prosecutions that members were no longer surprised at such statements. If they had heard the speaker say that lawyers were abandoning their profession from this cause, they would have been startled, but to us the fact is familiar.

"It is proper for us, then, to interrogate ourselves. Why is it that we are held to an accountability so much more strict than any other professional men, or than any other artizans? Is it because there are jealous and designing men our own ranks who instigate these suits? No doubt such men may be found, but only as an exception. The fact is that sometimes surgeons have been mulcted in damages simply because the jury believed, from the united character of the medical testimony, that it was a conspiracy, and the more conclusive the testimony, the more certain, with some jurors, is the defendant to suffer.

"Is it chargable to the members of another profession—to the lawyers? There may be some men in the profession of the law also, who, driven by the sheer necessity of the circumstances—by their extreme poverty—or who, without any such apology, with only loose notions of right and wrong, encourage and undertake such suits—such are the men who hang about the tombs in New York, and who may be found, more or less, in every town—but the speaker has reason to believe that honorable and intelligent lawyers seldom countenance these prosecutions. That eminent jurist of the State of New York, Joshua Spencer, has told Dr. Hamilton that for himself he does not think he ever commenced a suit of this character, although he has been frequently retained as counsel, and he believes his brethren, generally, look upon these complaints with suspicion and refuse to meddle with them.

"Where, then, must we look for an answer to the question, Why are these prosecutions against surgeons so frequent? Let the gentlemen be assured the causes are to be found in the *very imperfections of our art, and in our unwillingness to admit these imperfections.* Surgeons have claimed too much, and it cannot certainly be expected that the world will demand of them less than they claim for themselves. Again and again surgeons have said that the fracture of the femur might be generally made to unite without any shortening, while the fact is not so. Malgaigne, who is eminently an honest man, says, to make this bone unite in an adult person, where the fracture is sufficiently oblique to prevent the ends from supporting each other, is "simply impossible," (*simplement impossible.*)

"Let the profession be wiser in future and acknowledge that they cannot perform impossibilities."

